# Multimodal Imaging Reveals Labbé Vein Thrombosis Mimicking Subdural Hematoma: A Diagnostic Pitfall in Emergency Neuroimaging

**DOI:** 10.7759/cureus.83887

**Published:** 2025-05-11

**Authors:** Kouichi Asahi

**Affiliations:** 1 Internal Medicine and Pediatrics, Kohokuekimae Ohisama Clinic, Tokyo, JPN; 2 General Medicine and Radiology, Dokkyo Medical University Saitama Medical Center, Saitama, JPN

**Keywords:** cerebral venous thrombosis, cortical vein thrombosis, diagnostic pitfall, labbé vein thrombosis, multimodal imaging, subdural hematoma mimicker

## Abstract

Labbé vein thrombosis is a rare cause of cerebral venous thrombosis that may mimic more common intracranial hemorrhagic pathologies, such as acute subdural hematoma or cortical hemorrhage, on initial CT. This case involves a 20-year-old male who presented with an acute right temporal headache and a hyperdense subcortical lesion on CT. The thrombus measured approximately 65 HU. MRI revealed a T1 hyperintense thrombus in the vein of Labbé, and digital subtraction angiography confirmed venous occlusion. The patient was treated with anticoagulation and recovered without neurological deficit. This case highlights a diagnostic pitfall in emergency neuroimaging and emphasizes the value of multimodal imaging for accurate diagnosis.

## Introduction

Cerebral venous thrombosis (CVT) is a rare but potentially serious cause of stroke, accounting for approximately 0.5-1% of all cases, with higher incidence observed in younger patients, females, and those with hypercoagulable conditions or infection [1]. Among the various subtypes, thrombosis of the vein of Labbé, a major anastomotic vein that drains the lateral temporal lobe into the transverse sinus, is particularly uncommon and often underrecognized [2,3].

The clinical presentation of Labbé vein thrombosis is frequently nonspecific, commonly manifesting as an isolated headache or mild focal neurological symptoms. Importantly, venous congestion may lead to cortical edema, parenchymal hemorrhage, or even subarachnoid hemorrhage (SAH), particularly in cortical vein thrombosis. Boukobza et al. have described that CVT-related SAH is typically localized to the cerebral convexities, distinct from aneurysmal SAH patterns [4].

In emergency settings, noncontrast CT remains the first-line imaging modality due to its speed, availability, and utility in excluding more common pathologies such as intracerebral hemorrhage, infarction, or mass lesions [5]. However, CVT-related findings can be subtle and easily missed. In particular, Labbé vein thrombosis may present as a linear hyperdensity mimicking acute subdural hematoma (ASDH) or cortical hemorrhage [4,5], making clinical suspicion and follow-up imaging essential for timely diagnosis.

While advanced modalities such as susceptibility-weighted imaging (SWI) and flow-sensitive black-blood (FSBB) MRI have demonstrated high sensitivity for venous thrombi [6,7], these are not always available in acute settings. Conventional MRI and digital subtraction angiography (DSA) remain essential tools, especially when an unusual venous pattern or diagnostic ambiguity arises.

## Case presentation

A 20-year-old previously healthy man presented to the emergency department with a sudden-onset, severe headache localized to the right temporal region. He denied any history of trauma, infection, coagulopathy, or anticoagulant use. His vital signs were stable, and neurological examination revealed no focal deficits.

**Figure 1:**
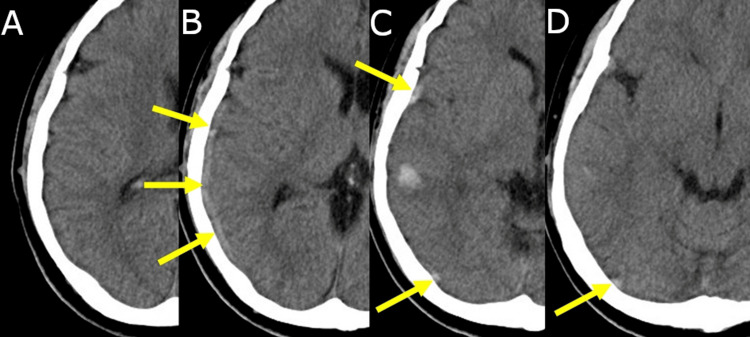
Noncontrast CT suggestive of ASDH along the right temporal convexity Initial noncontrast CT images (A-D) reveal a linear hyperdense area along the lateral convexity of the right temporal lobe (yellow arrows), which was initially interpreted as an ASDH with cortical hemorrhage. The hyperdensity is most prominent in slices B and C and remains visible through slice D. ASDH, acute subdural hematoma

An initial noncontrast CT scan revealed a curvilinear hyperdense structure (approximately 65 HU) running parallel to the sylvian fissure along the lateral convexity of the right temporal lobe, corresponding anatomically to the typical course of the vein of Labbé. No midline shift or significant mass effect was observed (Figure 1).

**Figure 2:**
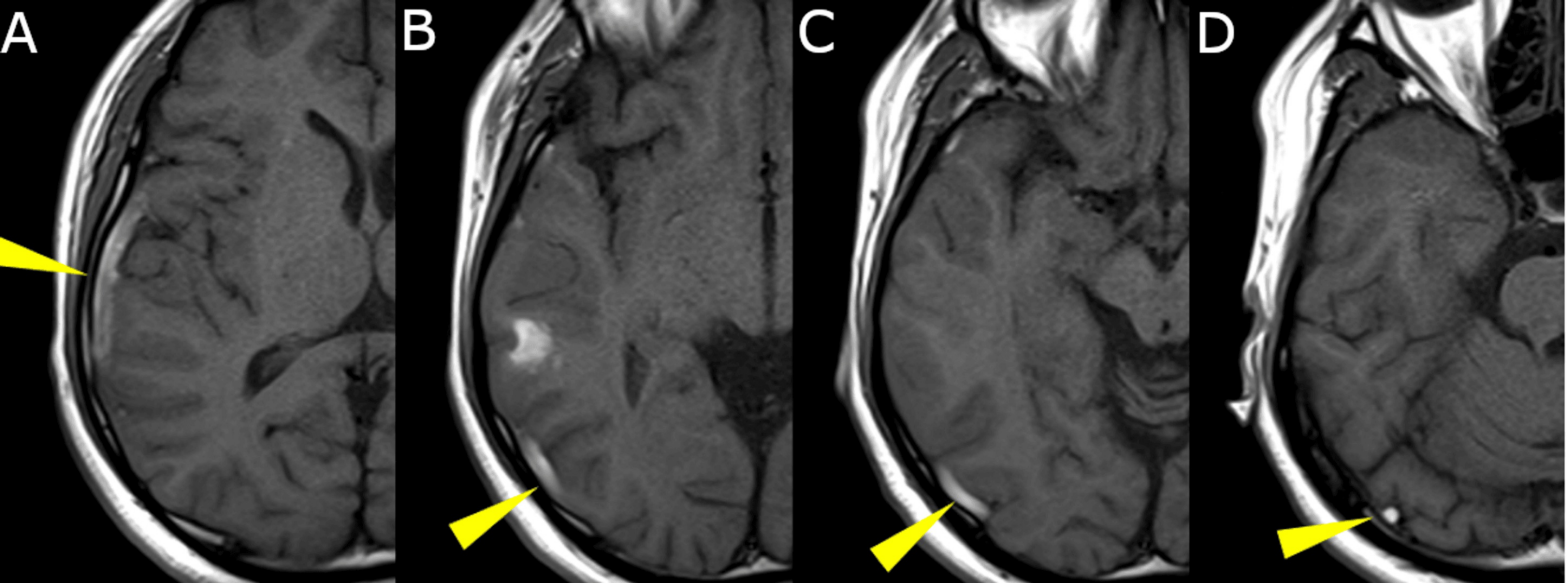
T1-weighted MRI revealing a linear hyperintense signal along the right vein of Labbé Axial T1-weighted MRI slices (A-D) demonstrate a continuous linear hyperintense signal along the anatomical course of the right vein of Labbé (yellow arrowheads), consistent with a subacute venous thrombus. The signal is most pronounced in slices A and B, corresponding to the right temporal convexity, and gradually attenuates posteriorly in slices C and D. In addition, a focal area of subcortical hyperintensity in slice B suggests a small subcortical hemorrhage, which may represent hemorrhagic transformation secondary to venous congestion.

MRI revealed a hyperintense tubular structure along the same trajectory on T1-weighted sequences, consistent with a subacute thrombus. Notably, there was no evidence of blooming artifact on gradient-echo sequences or restricted diffusion on DWI, which would indicate parenchymal hemorrhage or infarction. There was also no surrounding vasogenic edema or cortical swelling (Figure 2).

**Figure 3:**
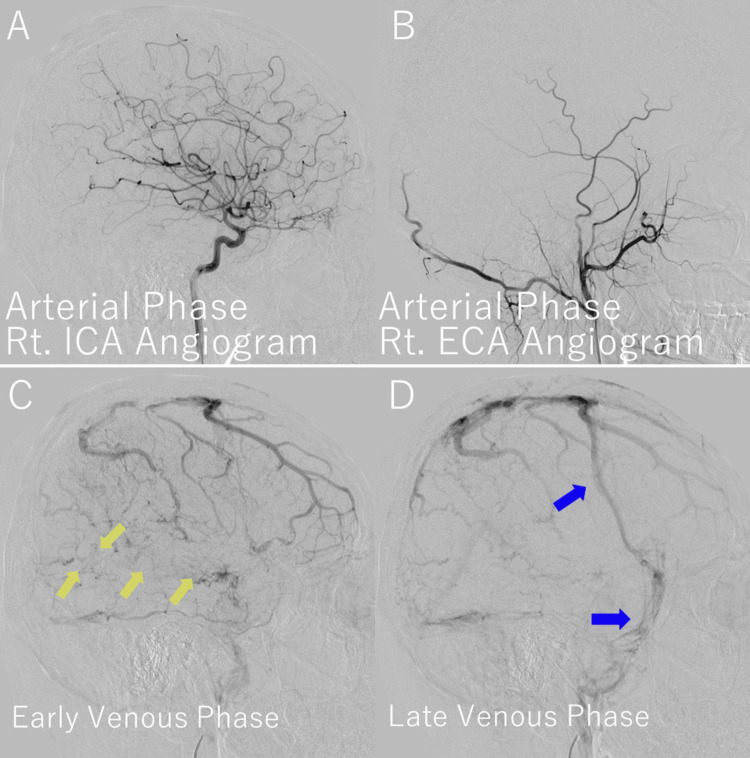
DSA demonstrating vein of Labbé thrombosis and collateral venous drainage (A) Arterial phase of the right ICA angiogram shows normal arterial anatomy without arteriovenous shunting. (B) Arterial phase of the right ECA angiogram is unremarkable. (C) Early venous phase reveals absence of contrast opacification along the expected course of the right vein of Labbé (yellow arrows), consistent with thrombosis. Surrounding fine cortical veins suggest early collateral recruitment. (D) Late venous phase shows persistent nonopacification of the Labbé vein, with blue arrows indicating collateral venous drainage through the vein of Trolard and the superficial middle cerebral vein, redirecting flow into the superior sagittal and transverse sinuses. DSA, digital subtraction angiography; ECA, external carotid artery; ICA, internal carotid artery

To confirm the diagnosis and delineate venous anatomy, cerebral angiography (DSA) was performed via right femoral artery access using a 5-Fr Simmons-type diagnostic catheter. A nonionic contrast agent was administered at 4 mL/s (total volume: 10 mL) into the right internal carotid artery. Biplanar imaging was used to assess venous outflow and collateral circulation. It demonstrated complete occlusion of the right vein of Labbé, with delayed filling and collateral venous drainage through posterior temporal and occipital cortical veins draining into the transverse sinus. No abnormalities in the arterial phase or dural sinuses were detected (Figure 3).

The patient was started on low-molecular-weight heparin for seven days. He remained neurologically intact throughout his hospital stay. Follow-up MRI at two weeks demonstrated partial recanalization, and by three months, complete resolution of the thrombus was observed. The patient remained symptom-free at the final outpatient follow-up, with a modified Rankin Scale of 0.

## Discussion

Labbé vein thrombosis is a rare subtype of cortical vein thrombosis, and its atypical location and nonspecific presentation make it particularly challenging to diagnose in the emergency setting. As demonstrated in this case, thrombosis of this vein may appear as a hyperdense, curvilinear lesion on noncontrast CT, mimicking ASDH or cortical hemorrhage. The “dense vein sign” observed here (65 HU) highlights the diagnostic ambiguity, particularly in young patients without trauma [5].

Importantly, compared to superior sagittal sinus thrombosis, which typically presents with diffuse intracranial hypertension, papilledema, and bilateral signs due to widespread venous drainage obstruction, Labbé vein thrombosis causes more focal and subtle symptoms, often limited to regional headache. This difference in both clinical and imaging presentation contributes to the underdiagnosis of LVT [3].

MRI proved invaluable in clarifying the diagnosis. The T1-weighted hyperintensity, in the absence of ischemia or diffusion restriction, supported a diagnosis of subacute thrombus. Although SWI and FSBB were not performed due to equipment limitations at the time, prior studies have shown their utility in detecting venous thrombi and microhemorrhages [6,7].

Importantly, no parenchymal or meningeal hemorrhage was evident in this case. Boukobza et al. have reported that CVT, particularly cortical vein involvement, can sometimes present with localized SAH, often confined to the cortical sulci of the convexity rather than basal cisterns [4]. In our case, such hemorrhagic findings were absent on both CT and MRI, allowing for safer anticoagulation.

DSA confirmed complete occlusion of the Labbé vein with evidence of collateral venous outflow. While DSA remains the gold standard for confirming venous patency due to its high spatial and temporal resolution, it is invasive. Noninvasive alternatives such as MR venography or CT venography may suffice in many settings and should be considered first when appropriate [8,9].

Treatment with anticoagulation led to full recovery, consistent with current recommendations. Although warfarin was not used, recent trials suggest that direct oral anticoagulants may be a safe and effective alternative in select CVT patients [10].

For emergency clinicians, we propose a simplified diagnostic approach: (1) consider venous thrombosis when CT shows atypical subcortical hyperdensity without trauma; (2) perform MRI with T1, DWI, and, if available, SWI or FSBB; and (3) reserve DSA for diagnostically ambiguous or anatomically complex cases.

This case underscores the importance of recognizing Labbé vein thrombosis as a distinct entity with diagnostic pitfalls. A high index of suspicion, anatomical knowledge, and multimodal imaging are key to timely diagnosis and optimal outcomes.

Although SWI was not included in the original protocol due to equipment limitations, and T2-weighted imaging showed no significant abnormalities such as cortical edema or signal dropout, we agree that their inclusion could enhance diagnostic confidence and uncover microhemorrhages or venous infarction. Future cases should incorporate these sequences when available, particularly in cases where hemorrhagic complications may influence treatment decisions.

## Conclusions

Labbé vein thrombosis should be considered in patients with unexplained temporal lobe hyperdensities on CT, particularly when trauma is absent. This case demonstrates that cortical vein thrombosis can mimic ASDH or even convexity SAH, leading to potential diagnostic errors. Multimodal imaging - including MRI and, when necessary, DSA - is essential to reach a definitive diagnosis. Early recognition and appropriate anticoagulation can result in excellent outcomes. Increased awareness of this condition among emergency clinicians and radiologists is critical to prevent misdiagnosis and unnecessary surgical intervention.
